# Region-Specific Differences in Amyloid Precursor Protein Expression in the Mouse Hippocampus

**DOI:** 10.3389/fnmol.2016.00134

**Published:** 2016-11-29

**Authors:** Domenico Del Turco, Mandy H. Paul, Jessica Schlaudraff, Meike Hick, Kristina Endres, Ulrike C. Müller, Thomas Deller

**Affiliations:** ^1^Institute of Clinical Neuroanatomy, Neuroscience Center, Goethe-UniversityFrankfurt, Germany; ^2^Institute of Pharmacy and Molecular Biotechnology (IPMB), Heidelberg UniversityHeidelberg, Germany; ^3^Clinic for Psychiatry and Psychotherapy, University Medical Center MainzMainz, Germany

**Keywords:** APP, dentate gyrus, CA1, immunostaining, western blotting, laser microdissection, *in situ* hybridization, RT-qPCR

## Abstract

The physiological role of amyloid precursor protein (APP) has been extensively investigated in the rodent hippocampus. Evidence suggests that APP plays a role in synaptic plasticity, dendritic and spine morphogenesis, neuroprotection and—at the behavioral level—hippocampus-dependent forms of learning and memory. Intriguingly, however, studies focusing on the role of APP in synaptic plasticity have reported diverging results and considerable differences in effect size between the dentate gyrus (DG) and area CA1 of the mouse hippocampus. We speculated that regional differences in APP expression could underlie these discrepancies and studied the expression of APP in both regions using immunostaining, *in situ* hybridization (ISH), and laser microdissection (LMD) in combination with quantitative reverse transcription polymerase chain reaction (RT-qPCR) and western blotting. In sum, our results show that APP is approximately 1.7-fold higher expressed in pyramidal cells of Ammon’s horn than in granule cells of the DG. This regional difference in APP expression may explain why loss-of-function approaches using APP-deficient mice revealed a role for APP in Hebbian plasticity in area CA1, whereas this could not be shown in the DG of the same APP mutants.

## Introduction

Amyloid precursor protein (APP) is an integral membrane protein involved in the pathogenesis of Alzheimer’s disease (AD). It is processed by proteases and cleaved into several biologically active fragments (e.g., Turner et al., 2003; Müller and Zheng, 2012; Zhang et al., 2012). Of note, proteolysis of APP by beta- and gamma-secretases generates the amyloid-ß (Aß) peptide, which oligomerizes, interferes with synaptic functions, and eventually aggregates into extracellular amyloid plaques, one of the neuropathological hallmarks of AD (Selkoe and Hardy, 2016). In contrast, proteolysis of APP by α-secretases (e.g., Postina et al., 2004; Yang et al., 2006; Fahrenholz, 2007; Prinzen et al., 2009; Saftig and Reiss, 2011; Kuhn et al., 2016), generates soluble APP-α (sAPPα), which is neuroprotective and important for neuronal plasticity (Turner et al., 2003; Ring et al., 2007; Aydin et al., 2012; Kögel et al., 2012). In the latter case, the Aß-peptide is not formed because α-secretases cleave APP within the Aß region of the protein. In AD the balance of this processing by secretases shifts towards the amyloidogenic pathway, which increases Aß production and leads to a lack of sAPPα (Endres and Fahrenholz, 2012) resulting in an impairment of cognition.

A region of the brain which is of particular interest in the context of AD is the hippocampus. Since the hippocampal formation and hippocampus-dependent learning and memory are affected early during the course of the disease (Braak and Braak, 1991) the hippocampus has been used as a model brain region to study the role of APP and its cleavage products in synaptic plasticity, learning and memory and neuroprotection (e.g., Turner et al., 2003; Ring et al., 2007). Interestingly, our physiological investigations of APP^−/−^ mice revealed remarkable differences between the subregions of the hippocampus: whereas APP was necessary for long-term potentiation (LTP) at the CA3–CA1 synapse (Ring et al., 2007; Weyer et al., 2011; Hick et al., 2015) it was not essential for LTP at the entorhinal cortex-granule cell (EC-GC) synapse in the dentate gyrus (DG; Jedlicka et al., 2012). We speculated that regional differences in basal APP expression or APP processing could explain these phenotypic differences. This interpretation would be in line with a recent publication, which reported APP to be predominantly expressed by interneurons in the DG (Wang et al., 2014).

To provide first evidence for this hypothesis and to reliably quantify differences in APP expression between granule cells of the DG and pyramidal cells of area CA1, we studied layer-specific expression levels of APP in the principal cell layers using laser microdissection (LMD) in combination with quantitative polymerase chain reaction (qPCR) and western blot analysis (e.g., Burbach et al., 2003; Del Turco et al., 2014). Since APP is alternatively spliced into three major isoforms (Kang et al., 1987; Tanzi et al., 1988; Sisodia et al., 1993; Rohan de Silva et al., 1997), i.e., APP-770, APP-695 and APP-751, assays detecting all major isoforms were employed. Furthermore, we used an antibody for western blotting, which is highly specific for APP and does not show staining on APP^−/−^ brain tissue (Guo et al., 2012) to quantify APP levels and to study its cellular distribution. The selection of the antibody appeared to be especially important, since some antibodies show unspecific background staining on tissue sections and may cross-react with APP-related proteins, such as the APP-like-proteins 1 or 2 (Anliker and Müller, 2006; Kaden et al., 2012; Müller and Zheng, 2012). Together with *in situ* hybridization (ISH) data for APP, our results show that APP is expressed exclusively by hippocampal neurons under physiological conditions. It is ~1.7 fold higher expressed by CA1 pyramidal cells compared to dentate granule cells, which may contribute to the regional differences seen in electrophysiological studies of APP^−/−^ mice (Ring et al., 2007; Jedlicka et al., 2012).

## Materials and Methods

### Animals

Adult (3–5 months old) male C57BL/6J mice (Janvier, France) and APP-deficient mice obtained from the colony at Heidelberg University (e.g., Li et al., 1996; Jedlicka et al., 2012) were used for experimental analysis. Animal care and experimental procedures were performed in agreement with the German law on the use of laboratory animals (animal welfare act; TierSchG). Animal welfare was supervised and approved by the Institutional Animal Welfare Officer.

### Immunofluorescence

Mice were deeply anesthetized with an overdose of pentobarbital (300 mg/kg body weight) and transcardially perfused with 0.9% sodium chloride (NaCl) followed by 4% paraformaldehyde (PFA) in phosphate-buffered saline (pH 7.4). Brains were removed, post-fixed for 4–24 h in 4% PFA and sectioned in the coronal plane (40 μm) using a vibratome (VT1000 S, Leica Microsystems). Free-floating sections were incubated in a blocking buffer containing 0.5% Triton X-100 and 5% bovine serum albumin (BSA) in 0.05 M Tris-buffered saline (TBS) for 30 min at room temperature followed by incubation in the primary antibody (diluted in 0.1% Triton X-100 and 1% BSA in 0.05 M TBS) overnight at 4°C. The following primary antibodies were used: mouse anti-APP (22C11, immunogen: 66–81 amino acids (aa) of purified recombinant Alzheimer precursor A4 fusion protein (N-terminus); MAB348, Chemicon), rabbit anti-APP (CT20, immunogen: synthetic peptide corresponding to 751–770 aa of human APP (C-terminus); 171610, Calbiochem), rabbit anti-APP (Y188, immunogen: synthetic peptide corresponding to C-terminus of human APP (YENPTY motif); ab32136, Epitomics), mouse anti-NeuN (A60, immunogen: purified cell nuclei from mouse brain; MAB377, Chemicon) and rabbit anti-GFAP (immunogen: GFAP isolated from cow spinal cord; Z0334, Dako). After several washes, sections were incubated with the appropriate secondary Alexa-conjugated antibodies (1:2000, Invitrogen, Waltham, MA USA) for several hours at room temperature, counterstained with Hoechst 33242 (Invitrogen) or DRAQ5 (Thermo Fisher Scientific, Waltham, MA, USA), and finally mounted in DAKO Fluorescent Mounting Medium (Dako).

### Western Blotting

For protein extraction, 10× volume of homogenization buffer (20 mM Tris, 500 mM NaCl, 0.5% CHAPS, 5 mM EDTA) was added to freshly dissected tissue samples, i.e., whole hippocampus as well as microdissected CA1 pyramidal cell layer (pcl) and dentate granule cell layer (gcl). Homogenization was performed with a pestle (Wheaton, Montgomery, MD, USA). After centrifugation at 4°C for 30 min (22,000 rpm, Sorvall WX Ultra Series, Thermo Electron Corporation), protein concentration was quantified with a Qubit^®^ 2.0 Fluorometer (Life Technologies, Carlsbad, CA, USA) using Qubit^®^ Protein Assay Kit (Life Technologies, Carlsbad, CA, USA). Samples were denatured for 5 min at 95°C and immediately cooled down on ice. For gel electrophoresis, protein amounts (approx. 30 μg for hippocampal tissue, 5–6 μg for microdissected tissue) were loaded onto 8% SDS–polyacrylamide gels and were separated at 120 V for 15 min followed by 160–180 V for 45 min. Subsequently, gels were blotted to nitrocellulose membranes at 15 V for 75 min. Blots were then washed twice in TBS and incubated with Odyssey Blocking Buffer (LI-COR Biosciences) at room temperature for 60–120 min. Blots were washed again in TBS and incubated overnight at 4°C with the appropriate primary antibody diluted in 1:1 Odyssey Blocking Buffer with TBS and 0.1% Tween20. Blots were washed in TBS with 0.1% Tween20 and incubated with an IRDye800CW conjugated secondary antibody (LI-COR Biosciences) at room temperature for 45 min. For normalization mouse anti-GAPDH antibody (Calbiochem) in combination with an IRDye680 conjugated goat anti-mouse antibody (LI-COR Biosciences) was used. Two-color imaging was performed using Odyssey^®^ Infrared Imaging System (LI-COR Biosciences). Densitometric analysis for each protein band was done using the Image Studio Software (LI-COR Biosciences). Each protein quantification was first normalized against GAPDH (loading control) from the same gel (intra-blot analysis), before comparisons for changes were made (inter-blot comparisons). The results (x-fold) are presented as means and standard deviations (SD) of three independent experiments. Statistics were analyzed using Student’s *t*-test. *P* values of ≤ 0.05 were considered statistically significant.

### *In situ* Hybridization

An ISH probe specific for all major *App* isoforms was designed to detect the juxtamembrane region of APP. To this end, a cDNA fragment encoding aa 492–623 of APP695 was cloned into the pcDNA3 vector. Prior to *in vitro* transcription, the plasmid was linearized and gel-purified using a gel extraction kit (Qiagen). *in vitro* transcription of DIG-labeled antisense RNA probe from the SP6 promoter was performed using the Roche DIG RNA labeling kit (SP6/T7), following the manufacturer’s instructions. Probes were subsequently purified using RNase-free ChromaSpin 100 columns (Clontech). The quantity of labeled and purified probe was estimated by Dot blot as described in the DIG RNA labeling kit manual.

Whole mouse brains were dissected and immediately placed on dry ice until they were thoroughly frozen. Brain slices (14 μm) were cut on a cryostat (Zeiss Hyrax C50), collected on Superfrost plus slides (Thermo Scientific) and dried at 56°C for 30 min. Sections were fixed for 10 min in 4% PFA in PBS, washed thrice in diethyl pyrocarbonate (DEPC)—treated PBS, and then permeabilized and acidified in triethanolamine hydrochloride (TEA-HCl)—acetic anhydride for 10 min. After three washing steps with DEPC-PBS, slices were dehydrated in an ethanol series (50%, 75%, 95%, 100%; 5 min each) and dried for at least 2 h at 56°C. Anti-sense probe was diluted in hybridization buffer to the final concentration of approx. 400 pg/μl and heated to 80°C for 10 min. After cooling down on ice, 100 μl of hybridization solution were applied to each slide, which was then covered with parafilm. Hybridization was done overnight at 56°C. On the next day, slides were placed in 4× SSC for 10 min to wash off excess probe. Stringent washing steps were 30 min in 0.2× SSC at 60°C, followed by another 90 min in fresh 0.2× SSC at 60°C, followed by 10 min in 0.2× SSC at room temperature. For probe detection, slides were equilibrated in P1DIG (100 mM Tris-HCl; 150 mM NaCl) for 10 min and blocked in blocking solution (P1DIG + 0.5% BSA + 1% Blocking reagent, Roche) for 30 min. Brain slices were encircled with PAP PEN and anti-DIG-AP antibody (80 μl, diluted 1:500 in blocking solution) was pipetted on every brain slice. Antibody incubation was done overnight at 4°C in a humidified chamber. The next day, all slides were washed twice for 15 min in P1DIG, then equilibrated in P3DIG (100 mM Tris-HCl; 100 mM NaCl; 50 mM MgCl2, pH 9.5) for 2 min. Slides were incubated in substrate solution (NBT/BCIP, diluted 1:50 in P3DIG) overnight at room temperature until color development was sufficient. Slides were then washed in PBS, fixed for 10 min in 4% PFA in PBS, washed in P4DIG (10 mM Tris-HCl; 1 mM EDTA, pH 8.0) for 10 min, then air dried for 2 h and finally mounted in Mowiol (Polysciences).

### Digital Illustrations

Figures were prepared digitally using commercially available graphics software (Photoshop, Adobe Inc., San Jose, CA, USA). Fluorescent images were acquired using a digital camera (Digital Sight DS-M5c, Nikon, Germany) or confocal microscopy (Eclipse C1 Plus, Nikon). Single fluorescent images of the same section were digitally superimposed. The contrast, brightness and sharpness of images were adjusted as needed for each section. No additional image alteration was performed.

### Laser Microdissection

Mice were killed by an overdose of isoflurane (Abbott). Brains were rapidly removed from the cranium, embedded in tissue freezing medium and immediately flash-frozen in −70°C isopentane cooled by dry ice. Cryostat sections (8 μm for RNA analysis, 20 μm for western blotting) were mounted on polyethylene naphthalene (PEN) or polyester (POL) slides (Leica Microsystems). For RNA analysis, sections were fixed shortly in −20°C cold acetone, stained with 1% cresyl violet staining solution and dehydrated in 75% and 100% ethanol. Using a Leica LMD6500 system (Leica Microsystems), defined tissue samples of the dentate gcl and of CA1 pcl were collected separately from the same brain sections and transferred to −80°C until further processing.

### RNA Isolation and Reverse Transcription

Total RNA was isolated using the RNeasy Plus Micro Kit (Qiagen) according to the manufacturer’s recommendations. RNA integrity was assessed using the Agilent 2100 Bioanalyzer system and Agilent RNA 6000 Pico Kit (Agilent Technologies), and then reverse transcribed using High Capacity cDNA Reverse Transcription Reagents Kit (Applied Biosystems) following the manufacturer’s recommendations.

### Quantitative Polymerase Chain Reaction (qPCR)

cDNA was amplified using TaqMan^®^ Fast Universal PCR Master Mix (Applied Biosystems) and the StepOnePlus Real-Time PCR System (Applied Biosystems). PCR products were checked on Agilent DNA 1000 Chips (Agilent Technologies) with the Agilent 2100 Bioanalyzer system to verify product specificity and amplicon size. Quantification of the gene expression of candidate reference genes was carried out using SYBR^®^ GreenER^TM^ qPCR Supermix Universal (Invitrogen, Waltham, MA, USA) following the manufacturer’s recommendations. Primer efficiencies and quantification cycle (Cq) values were calculated using LinRegPCR Software (Tuomi et al., 2010). To determine the most stable reference genes and the minimum number for accurate normalization, NormFinder (Andersen et al., 2004) and geNorm (Vandesompele et al., 2002) were used according to the developer’s manuals. qPCR data were tested for statistical significance using one-way ANOVA followed by Bonferroni *post hoc* test to correct for multiple comparisons, *p ≤ 0.05.

## Results

### APP is Differentially Expressed in the Principal Cell Layers of the Hippocampus

**Figure 1:**
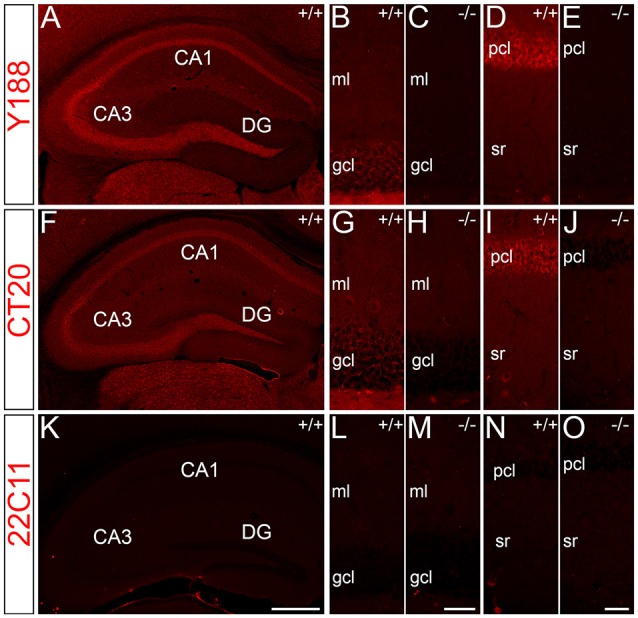
**Specificity of amyloid precursor protein (APP) antibodies tested on hippocampal sections of adult wild type and APP deficient mice. (A)** Immunofluorescence of the dorsal hippocampus of wild type (^+/+^) mice using the Y188 antibody. The dentate gyrus (DG) shows only a weak signal, whereas a more intense labeling is seen in Ammon’s horn (CA1–3). **(B,C)** Immunofluorescence is detectable in the granule cell layer (gcl) and molecular layer (ml) of the DG in wild type but not in brain sections of APP deficient mice using the Y188 antibody. **(D,E)** Principal cell layer (pcl) of CA1 shows a strong signal in wild type mice. Some immunofluorescence is also seen in stratum radiatum (sr). In contrast, staining is absent in APP-deficient hippocampal tissue. **(F–J)** Immunofluorescence of the hippocampus of wild type and APP deficient mice using the CT20 antibody. **(F,G,I)** Similar to the results with Y188 a stronger signal can be seen in Ammon’s horn (CA1–3) of wild type mice compared to the DG. **(H,J)** Background staining is slightly higher in APP-deficient tissue sections compared to the background seen with the Y188 antibody (in **C,E**). **(K–O)** Immunostaining using the 22C11 antibody shows similar staining in wild type and APP-deficient tissue, suggesting that this antibody is not sufficiently specific to identify APP in tissue sections **(K)**, DG **(L,M)** and CA1 **(N,O)**. Scale bars: **(K)** 500 μm; **(M,O)** 25 μm.

For immunohistochemical detection of APP protein in the adult mouse hippocampus, widely used antibodies against APP were selected which recognize the major isoforms of APP in the rodent brain, i.e., APP-770, APP-695 and APP-751. To address the specificity of these antibodies, we tested the antibodies on wild type (APP^+/+^) and APP deficient (APP^−/−^) brain tissue sections. Two of the antibodies, i.e., Y188 and CT20, that both recognize C-terminal APP epitopes showed immunoreactivity only in APP^+/+^ brain sections but virtually no staining in APP^−/−^ hippocampal tissue (Figures 1A–J). Using these antibodies, a considerably stronger fluorescence signal was observed in the principal layers of the Ammon’s horn compared to the dentate gcl (Figures 1A,B,D,F,G,I). Non-specific immunoreactivity was moderately higher in APP^−/−^ sections using CT20 compared to the Y188 antibody (Figures 1C,E,H,J). In contrast, the 22C11 antibody did not show specific staining (Figures 1K–O).

**Figure 2:**
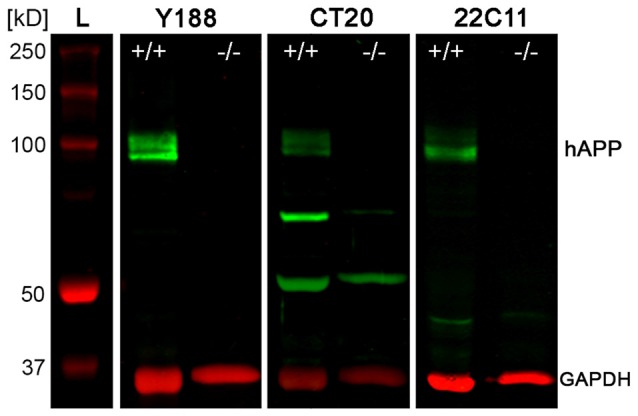
**Western blot analysis of APP protein in adult mouse hippocampus.** Western blotting of the three antibodies used for detecting APP, i.e., Y188, CT20 and 22C11, shows the typical set of bands corresponding to holoAPP (hAPP) protein (~95–100 kDa) in hippocampal homogenates of adult wild type (APP^+/+^) mice but not in APP deficient (APP^−/−^) tissue. Additional bands of smaller sizes seen with both CT20 and 22C11 antibodies are not specific for APP, as they are also seen in the APP KO control. GAPDH (~35–38 kDa) was used as loading control. L: Ladder (Precision Plus Protein™ Dual Color Standards, BIO-RAD).

**Figure 3:**
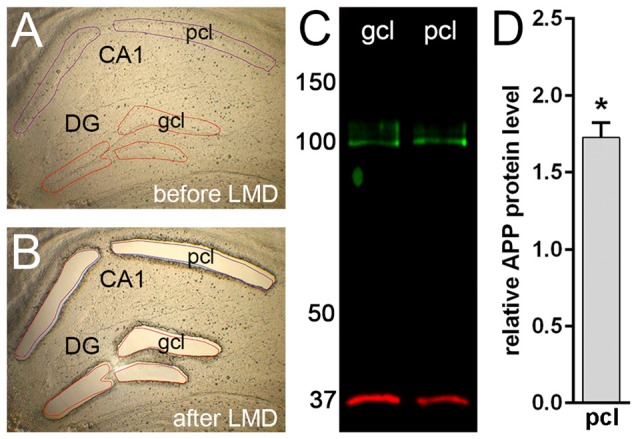
**Western blot analysis of APP protein in microdissected hippocampal subregions of adult mouse. (A,B)** Laser microdissection (LMD) of hippocampal subregions, i.e., CA1 pyramidal cell layer (pcl) and the gcl of the DG. **(C)** Western blotting using the Y188 antibody shows a specific signal corresponding to APP protein (~95–100 kDa) in DG gcl and CA1 pcl. GAPDH (~35–38 kDa) was used as loading control. **(D)** Quantitative western blot analysis reveals higher APP protein levels (~1.7-fold) in CA1 pcl relative to DG gcl samples. Data (*N* = 3 mice, *n* = 3 for each region) were tested for statistical significance using *t*-test (two-tailed), *p ≤ 0.05. Values are represented as mean ± standard deviations (SD).

To quantify protein levels and to corroborate our immunofluorescence data, we performed double-fluorescence western blot analysis using whole hippocampal homogenates as well as laser microdissected tissue samples of CA1 pcl and DG gcl. Holo-APP (~95–100 kDa) was recognized by all three APP antibodies in wild type but not in APP deficient tissue samples (Figure 2). CT20 and 22C11 demonstrated additional fragments of smaller size in both genotypes (Figure 2). The Y188 antibody appeared to be the most specific of the three, which was in line with our recent western blot results using this antibody indicating that it primarily detects full-length APP whereas the relative abundance of C-terminal stubs that are detected by this antibody is much lower (Fol et al., 2016). Based on these results, we chose Y188 to quantitatively determine APP in laser microdissected samples of hippocampal subregions (Figure 3).

In line with our immunofluorescence labeling, quantitative western blot analysis of microdissected tissue revealed a significantly higher APP protein level (approximately 1.7 fold) in the pcl of CA1 compared to the gcl of the DG (Figure 3). These data confirmed our initial impression that APP is differentially expressed in these two hippocampal subregions.

### APP is Predominantly Expressed by Neurons in the Adult Mouse Hippocampus

**Figure 4:**
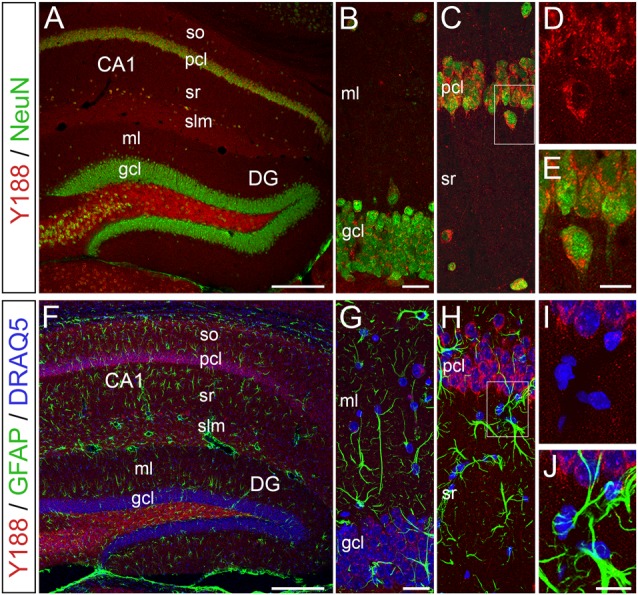
**APP immunoreactivity is predominantly detected in neurons of the adult mouse hippocampus. (A–E)** Confocal double-immunofluorescence staining for APP using Y188 (red) in combination with the neuron-specific marker NeuN (green) in wild type brain sections shows a strong neuronal APP expression in the pcl of area CA1 compared to the DG gcl. of note, Y188 and NeuN double positive cells were also found in adjacent layers (e.g., hilus and stratum radiatum, sr). **(F–J)** Double-immunofluorescence staining for APP (Y188, red) and the astrocytic marker GFAP (green) revealed no APP expression by this glial cell population in the adult mouse hippocampus. DRAQ5 was used to visualize cell nuclei (blue). Scale bar: **(A,F)** 200 μm; **(B,G)** 25 μm; **(E,J)** 12.5 μm.

To elucidate, which hippocampal cell types produce relevant amounts of APP protein, we performed confocal double-immunofluorescence analysis using Y188 in combination with the neuron-specific marker NeuN (neuronal nuclear antigen) or the astrocytic marker GFAP (glial fibrillary acidic protein; Figure 4). We performed this staining since earlier publications, which were in part performed in tissue cultures, had also suggested an astroglial expression of APP (Golde et al., 1990; Haass et al., 1991; LeBlanc et al., 1991). In our preparations, we found that APP is predominantly expressed by hippocampal neurons (Figures 4A–D). In contrast, we did not detect an astroglial APP expression (Figures 4F–J). Of note, APP-positive neurons were not only found in the principal cell layers of the hippocampus, i.e., in pcl of Ammon’s horn and, to some weaker extent, in the dentate gcl, but also in adjacent layers, e.g., hilus, stratum radiatum (sr) or stratum lacunosum-moleculare (slm; Figures 4A,C).

**Figure 5:**
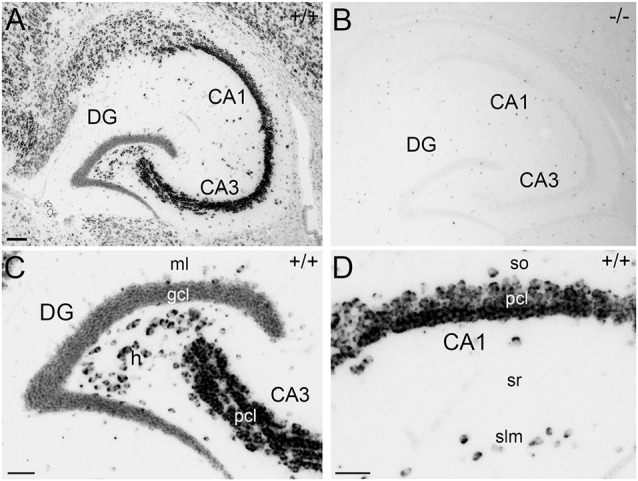
**Non-radioactive *in situ* hybridization (ISH) for *App* mRNA in the adult mouse hippocampus. (A)** ISH using a digoxygenin-labeled anti-sense riboprobe demonstrates App mRNA expression in the hippocampus of an adult wild type (APP^+/+^) mouse. Principal layers of Ammon’s horn show a strong expression for *App* mRNA. **(B)** Hippocampal tissue of APP deficient (APP^−/−^) mice served as negative control and was stained with the same anti-sense riboprobe. This showed only non-specific signals. **(C)** *App* mRNA expression is strong in the pcl of area CA3 and in the dentate hilus (h). In contrast, the mRNA-signal is much weaker in the gcl of the DG. Molecular layer (ml). **(D)** *App* mRNA is strongly expressed in pcl of CA1. In addition, a small number of *App* mRNA—positive cells are found in the adjacent layers, e.g., stratum oriens (so), stratum radiatum (sr), or stratum lacunosum-moleculare (slm). Scale bars: **(A)** 100 μm; **(C,D)** 50 μm.

To also identify *App* mRNA-expressing cells in the hippocampus, we next performed non-radioactive ISH using a digoxygenin-labeled riboprobe. This probe detects an mRNA sequence corresponding to the juxtamembrane region of APP, which is present in all major APP isoforms, i.e., APP-770, APP-751 and APP-695, but not conserved in the related APLPs. Strongly *App* mRNA-expressing cells were detected in the pcl of Ammon’s horn and in the hilus of the DG, whereas only a comparatively weak ISH signal was observed in the dentate gcl (Figures 5A,C). Outside the principal cell layers, only few *App* mRNA-expressing cells could be found in the adjacent layers, e.g., sr or slm (Figure 5D), which is an expression pattern that corresponds to the expression of APP by interneurons (Wang et al., 2014) but not by astroglia. Hippocampal tissue of APP deficient mice served as negative control and was stained using the same anti-sense riboprobe. This experiment revealed only weak non-specific background (Figure 5B).

Together, the results obtained by ISH, immunofluorescence and western blot analysis suggest that *App* mRNA as well as APP protein are predominantly expressed by principal neurons but not by astroglial cells in the adult mouse hippocampus.

### Quantitative Analysis of *App* mRNA Expression in Hippocampal Subregions

**Figure 6:**
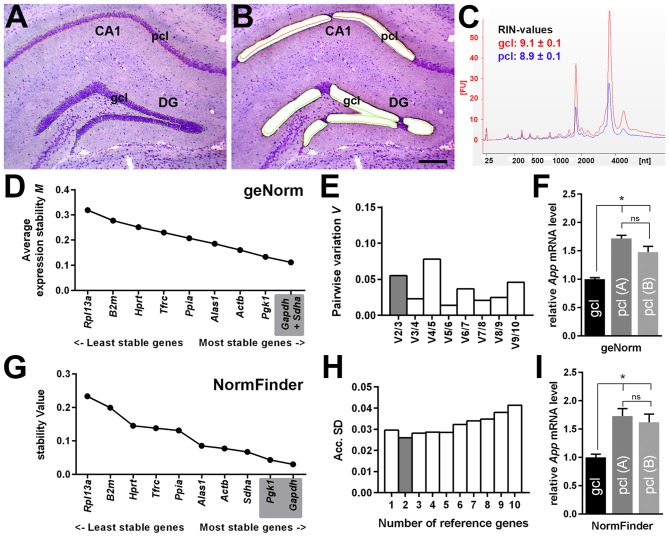
**Quantitative *App* mRNA expression in hippocampal subregions. (A,B)** LMD of hippocampal subregions, i.e., CA1 pcl and gcl of the DG. Representative section of the dorsal hippocampus (coronal plane, cresyl violet staining) before **(A)** and after **(B)** LMD is shown. Scale bar: 250 μm. **(C)** RNA integrity analysis of total RNA isolated from the dissected gcl (red) and from pcl (blue) demonstrating highly intact RNA (RIN-values: ~9; Agilent 2100 Bioanalyzer). **(D,E)** Average expression stability values (*M*) and evaluation of the optimum number of candidate reference genes for CA1 pcl and for DG gcl according to geNorm software. Pairwise variation (V) of candidate reference genes indicates that the use of the two most stable genes is sufficient to obtain an accurate normalization index for quantitative PCR (qPCR) analysis. **(F)** A significantly higher gene expression for *App* (1.5- to 1.7-fold) was detected in CA1 pcl relative to DG gcl using two different *App*-specific TaqMan assays (**A**: Mm_01344172_m1, **B**: Mm_00431830_m1) after normalization to a reference gene index calculated by geNorm. **(G,H)** Gene expression stability values (*S*) and accumulated SD analysis using NormFinder. The minimal number of reference genes required for effective normalization is highlighted. **(I)** Comparable to the results obtained by geNorm algorithm, a significantly higher *App* expression (1.6- to 1.7-fold) was detected in CA1 pcl relative to DG gcl after normalization to the reference gene index estimated by NormFinder. Data (*N* = 5–6 mice) were tested for statistical significance using one-way ANOVA followed by Bonferroni *post hoc* test to correct for multiple comparisons, *p ≤ 0.05.

By using qPCR in combination with LMD, we aimed to compare *App* mRNA expression levels in the pcl of CA1 compared to the gcl of the DG. For this purpose, only high quality RNA samples (RIN-values: ~9) of laser microdissected cell layers were used (Figures 6A–C). To more reliably analyze possible differences in gene expression, we first validated a panel of suitable reference genes (see Table 1 for details) for both hippocampal subregions in order to achieve robust qPCR data. Two established and widely accepted algorithms, i.e., geNorm and NormFinder, were used for the expression stability ranking of reference genes for CA1 and DG (Table 2). As determined by pairwise variation using geNorm and accumulated SD analysis according to NormFinder, the most stable reference genes as well as the minimal number necessary for accurate normalization were determined (Figures 6D,E,G,H). Of note, both algorithms showed a comparable ranking for all of the candidate reference genes tested (Table 2; Figures 6D,G).

**Table 1:** **Details of qPCR assays used in this study**.

Gene symbol	Gene name	Accession number	TaqMan assay number	Exon	Location	Amplicon size (bp)
*App*	amyloid beta (A4) precursor protein	NM_001198823.1	Mm01344172_m1	17–18	2358	111
		NM_001198824.1		16–17	2301
		NM_001198825.1		15–16	2247
		NM_001198826.1		16–17	2304
		NM_007471.3		15–16	2133
*App*	amyloid beta (A4) precursor protein	NM_001198823.1	Mm00431830_m1	14–15	2064	82
		NM_001198824.1		13–14	2007
		NM_007471.3		12–13	1839
*Gapdh*	glyceraldehyde-3-phosphate dehydrogenase	NM_008084.3	Mm99999915_g1	2–3	265	107
		NM_001289726.1		2–3	117
*Pgk1*	phosphoglycerate kinase 1	NM_008828.3	Mm00435617_m1	5–6	675	137
*Sdha*	succinate dehydrogenase complex, subunit A, flavoprotein (Fp)	NM_023281.1	Mm01352366_m1	6–7	804	82

**Gene symbol**	**Gene name**	**Accession number**	**Primers (forward, reverse)**	**Exon**	**Location**	**Amplicon size (bp)**

*Actb*	actin, beta	NM_007393.3	GAAGATCAAGATCATTGCTCCT TGGAAGGTGGACAGTGAG	5–6	1054–1137	84
*Alas1*	aminolevulinic acid synthase 1	NM_020559	CGATGCCCATTCTTATCC TTGAGCATAGAACAACAGAG	2	210–284	75
*B2m*	beta-2 microglobulin	NM_009735	CCTCTGTACTTCTCATTACTTG GCCTCTTTGCTTTACCAA	4	670–761	92
*Hprt*	hypoxanthine guanine phosphoribosyl transferase	NM_013556.2	GTGATTAGCGATGATGAAC TTCAGTCCTGTCCATAATC	2–3	988–1065	117
*Gapdh*	glyceraldehyde-3-phosphate dehydrogenase	NM_008084.2	ACAATGAATACGGCTACAG GGTCCAGGGTTTCTTACT	7	171–287	78
*Pgk1*	phosphoglycerate kinase 1	NM_008828.2	CGTGATGAGGGTGGACTT TGGAACAGCAGCCTTGAT	1–3	184–262	79
*Ppia*	peptidylprolyl isomerase A	NM_008907.1	CAAGACTGAATGGCTGGAT ATGGCTTCCACAATGTTCA	4–5	392–466	75
*Rpl13a*	ribosomal protein L13A	NM_009438.5	TCCACCCTATGACAAGAA GTAAGCAAACTTTCTGGTAG	5–7	348–432	85
*Sdha*	succinate dehydrogenase complex, subunit A, flavoprotein (Fp)	NM_023281.1	CAAGACTGGCAAGGTTAC ATCAGTAGGAGCGGATAG	14–15	1928–2028	101
*Tfrc*	transferrin receptor	NM_011638	CTATATCGGAGACAGTGAT GCTACAGGAAGTTAGGAA	19	307–433	148

**Table 2:** **Ranking of candidate reference genes by geNorm and NormFinder**.

CA1 (pcl) + DG (gcl)				
Rank	geNorm (*M*)		NormFinder (*S*)	
1	*Gapdh + Sdha*	0.111	*Gapdh*	0.030
2			*Pgk1*	0.043
3	*Pgk1*	0.133	*Sdha*	0.067
4	*Actb*	0.160	*Actb*	0.077
5	*Alas1*	0.185	*Alas1*	0.085
6	*Ppia*	0.207	*Ppia*	0.131
7	*Tfrc*	0.230	*Tfrc*	0.138
8	*Hprt*	0.251	*Hprt*	0.145
9	*B2m*	0.277	*B2m*	0.199
10	*Rpl13a*	0.319	*Rpl13a*	0.233

*Expression stability values of candidate reference genes for CA1 pyramidal cell layer (pcl) and for granule cell layer (gcl) of the dentate gyrus (DG) calculated by geNorm (*M*-values) and NormFinder (*S*-values) algorithms*.

Based on this data set, we used a normalization index out of the two most stable reference genes as well as the best combination of suitable genes, i.e., *Gapdh* and *Sdha* for geNorm, and *Gapdh* and *Pgk1* for NormFinder, respectively. For *App* gene expression analysis, two different qPCR assays specific for all major *App* isoforms were selected, which detected almost identical gene expression levels. Using this strategy, we determined a significantly higher expression of *App* mRNA (1.5- to 1.7-fold) in CA1 pyramidal cells compared to granule cells of the DG using either of the reference gene indices for accurate normalization of qPCR data (Figures 6F,I).

## Discussion

In the present study, we analyzed the expression of APP at the protein and mRNA level in the gcl and CA1 pcl of the adult mouse hippocampus using confocal immunofluorescence, ISH and LMD in combination with qPCR or western blot analysis. Our main findings can be summarized as follows: full-length APP is expressed by neurons under physiological conditions. APP expression is ~1.7× stronger at both mRNA and protein level in CA1 pyramidal cells compared to dentate granule cells. We propose that these differences in basal APP expression may contribute to the regional differences in APP function we reported in earlier studies using APP^−/−^ animals (Ring et al., 2007; Jedlicka et al., 2012).

### Endogenous Full-Length APP Levels in the Mouse Hippocampus—Methodological Considerations

Quantification of endogenous APP levels in the brain is confounded by the fact that some commercially available antibodies recognize not only full-length APP but also APP cleavage products and/or other protein fragments (Guo et al., 2012). In addition, antibodies may cross-react with the highly homologous APLPs, which further limits antibody specificity (Slunt et al., 1994). Thus, we ensured using tissue of APP^−/−^ mice that the antibodies we used for immunohistochemistry and western blot analysis in this study are highly specific and can be employed to detect holo-APP in the mouse hippocampus with high reliability. Furthermore, since APP is expressed in different isoforms in the nervous system and the brain (Kang and Müller-Hill, 1990; Sisodia et al., 1993), we designed probes for ISH and primers for qPCR which detect the three major isoforms of APP. Choice of these tools for our quantitative analysis make us confident that we predominantly measured total full-length APP mRNA and protein in our study.

Furthermore, since we were specifically interested in the neuronal expression of APP in these two regions and since our immunohistochemistry revealed a neuron-specific expression pattern of APP in the hippocampus (see below) we used LMD to selectively harvest the neuronal cell layers, i.e., the gcl of the DG and the CA1 pcl, respectively. This approach makes our quantification quite specific for granule cells and CA1 pyramidal cells, since the number of principal cells by far exceeds the number of cells of other cell types in these layers. Thus, we are confident that we here report robust and reliable data on the relative expression of APP mRNA and protein in the principal neurons of two major subfields of the hippocampus.

### Full-Length APP is Expressed by Neurons in the Mouse Hippocampus

In the adult rodent CNS, three major APP isoforms encoded by alternatively spliced transcripts have been described. In line with Guo et al. (2012), our data indicate that in tissue of intact and otherwise untreated mouse brain endogenous APP is expressed selectively by neurons but not astroglia: neither immunostaining with the APP-specific antibody Y188 nor ISH with *App*-specific riboprobes revealed a glial expression pattern. Similarly, double-labeling for neuronal and astroglial markers revealed a highly selective neuronal expression. In culture, however, previous studies reported that both astrocytes and microglia express APP (Haass et al., 1991; LeBlanc et al., 1991; Forloni et al., 1992; Mönning et al., 1995) and during aging Aß production has also been reported from non-neuronal sources in transgenic APP overexpressing mice. Thus, the possibility exists that glial cells express APP under reactive conditions *in vivo*. This issue was previously addressed by Guo et al. (2012), who used a traumatic brain injury model and an AD mouse model and failed to detect APP-positive astrocytes using APP-specific antibodies. They concluded that *in vivo* APP levels in astrocytes may be too low for detection even under reactive conditions (Guo et al., 2012). In our own investigations, in which we used entorhinal cortex lesions (Lynch et al., 1978; Steward, 1994; Deller and Frotscher, 1997) to denervate the DG, we also failed to see an increase in *App* mRNA in the denervated outer molecular layer (Del Turco et al., 2014). In this layer, reactive glia are particularly abundant (Deller et al., 2000, 2007; Del Turco et al., 2014). Although these two reports cannot rule out the possibility that under some other conditions APP is expressed *in vivo* by glial cells, they certainly suggest that glial APP is not the primary source of APP in the intact or injured brain.

### Endogenous Neuronal *App* mRNA Levels are Tightly Controlled

It has been pointed out by others that *App* is regulated very much like a housekeeping gene (Dawkins and Small, 2014). The fact that the *App* promoter lacks TATA and CAAT boxes but contains sites for several transcription factors regulating the expression of proteins associated with cell proliferation and differentiation suggests that *App* mRNA levels are primarily regulated during development (Izumi et al., 1992; Clarris et al., 1995; for review see Dawkins and Small, 2014). In the adult brain *App* mRNA levels may be much more tightly controlled to supply neural tissue with a constant level of APP protein for further processing.

However, a certain degree of transcriptional regulation has been reported for APP and *App* mRNA in adult neurons following brain injury (Murakami et al., 1998; Van Den Heuvel et al., 1999, 2007; Itoh et al., 2009). This appears to be of relevance, since head trauma is considered a risk factor for AD (e.g., Mortimer et al., 1991; Szczygielski et al., 2005). Concerning this lesion-induced regulation, the experimental literature is somewhat controversial (Szczygielski et al., 2005). By hindsight this is not surprising since many different antibodies and probes were used and some of them may not have been tested for specificity. In our own investigations using the entorhinal cortex lesion model we initially failed to observe an increase in *App* mRNA using screening methods. Only after using the sensitive LMD/qPCR approach (Burbach et al., 2003), which made it possible to measure *App* mRNA within microdissected cell and fiber layers did we detect a ~1.3-fold increase of *App* mRNA in denervated granule cells at 7 days post lesion (Del Turco et al., 2014). We conclude that neuronal *App* expression is tightly regulated and even under extreme conditions, e.g., brain injury, *App* gene expression changes range between 1- to 2-fold. If translated 1:1 into protein, as our present study suggests, such an increase in *App* mRNA may, however, be biologically and pathophysiologically relevant.

Finally, it should be kept in mind that transcriptional regulation of *App* is only one regulatory step under physiological and pathophysiological conditions, likely limiting the amount of full-length APP protein available for downstream processing. Post-transcriptional regulation by miRNAs has also been recently described (Schonrock et al., 2012). Most importantly, however, the amount, availability and activity of the secretases eventually decide the “biological fate” of the full-length protein by liberating its biologically active fragments. In contrast to the levels of *App* mRNA, which appear to be tightly controlled and provide neurons with a basal supply of APP, the activity and/or expression of secretases is regulated by neuronal activity and many other conditions, which have been reviewed elsewhere (Endres and Fahrenholz, 2012; Sun et al., 2012; Vassar et al., 2014; Vincent, 2016).

### Basal Expression of APP is Higher in CA1 Pyramidal Neurons Compared to Dentate Granule Cells

ISH against endogenous *App* mRNA revealed a weaker labeling of dentate granule cells compared to the pyramidal cells of Ammon’s horn. This made us wonder whether this reflected a true regional difference between the DG and the other hippocampal subfields. Since non-radioactive ISH cannot be reliably used for quantitative analysis, we quantified *App* mRNA using qPCR. Using LMD, the principal cell layers were harvested, which reduced dilution effects. The careful selection of reference genes using current recommendations for qPCR (Vandesompele et al., 2002; Andersen et al., 2004) ensured a very robust reference for the subregional comparison. In sum, this revealed a 1.5- to 1.7-fold difference in *App* mRNA expression between the DG and area CA1. The difference in *App* mRNA level translates into protein, since we used the same LMD approach to obtain the tissue for western blot analysis and found a comparable difference for APP protein, i.e., ~1.7-fold more protein in area CA1 compared to the DG.

### Regional Differences in APP Expression May Contribute to Regional Differences in Synaptic Plasticity

The physiological role of APP has been investigated in the hippocampus using APP^−/−^ mice. This loss-of-function approach revealed a robust role for APP in synaptic plasticity at the CA3-CA1 synapse (Dawson et al., 1999; Ring et al., 2007). Animals lacking APP showed an impaired LTP, which went hand-in-hand with memory dysfunctions. In contrast, using similar stimulation protocols for synaptic strengthening, the same line of APP^−/−^ mice did not show an LTP-defect at the EC-GC synapse *in vivo* (Jedlicka et al., 2012). This was a somewhat surprising result and we suggest—based on the data reported in this article—that differences in APP expression level between the two regions might contribute to the functional differences seen in our recordings.

How could different APP levels in neurons contribute to differences in synaptic function? Although the physiological role of APP is not yet fully understood several recent publications have suggested important functions for APP and its cleavage products at central synapses. With regard to full-length APP, it has been shown that it can act as a cell-adhesion molecule in *trans*, i.e., linking pre- and postsynapse, thus affecting the stability of synapses (Soba et al., 2005; Stahl et al., 2014). On the presynaptic side, APP regulates the abundance of synaptic vesicle proteins and may impact on synaptic transmission (Laßek et al., 2013, 2014, Laßek et al., 2016a,b; Fanutza et al., 2015). This presynaptic effect is in line with our own findings, which indicate that lack of APP causes presynaptic changes at the EC-GC (Jedlicka et al., 2012) as well as the CA3-CA1 synapses (Hick et al., 2015). On the postsynaptic side, sAPPα, which is generated from APP by α-secretase cleavage, appears to be required for synaptic strengthening. Experiments using sAPPα-binding antibodies and recombinant sAPPα (Turner et al., 2003; Taylor et al., 2008) as well as our own approaches using mouse genetics (Ring et al., 2007; Hick et al., 2015) revealed an essential function of this fragment in Hebbian-plasticity at both synapses. Most likely, the APP effect on synaptic plasticity is caused by an increased delivery of NMDAR to synapses (Cousins et al., 2009; Hoe et al., 2009), resulting in increased NMDAR currents (Taylor et al., 2008). In conclusion, APP and its cleavage products influence synaptic function at both pre- and postsynapse. It is thus highly likely that regional differences in APP levels could impact on the effect size experimenters can observe using APP-KO mice.

Unraveling and understanding the role of APP at central synapses is non-trivial and may ultimately require synapse-specific answers. In addition to the above discussed differences in APP levels, differences in APP processing and thus the abundance of specific fragments such as sAPPα between brain regions may also play an important role. Likewise, regional differences in the expression of APP-like proteins, i.e., APLP1 or APLP2, which can partially compensate for a loss of APP could affect the interpretation of loss-of-function experiments (von Koch et al., 1997; Heber et al., 2000; Weyer et al., 2011; Hick et al., 2015; Vnencak et al., 2015). Regardless of all these considerations, however, APP can only play a role in synaptic plasticity of a synapse if it is present. If not, other factors will predominate. Thus, we feel confident to conclude that APP plays a greater role for synaptic plasticity in area CA1 compared to the DG in mice. This finding, which implies that some effects of APP are region-specific, may be of relevance for future studies on APP and may also affect the design and analysis of APP-related animal models of AD.

## Author Contributions

DDT, MHP, JS, and MH performed experiments. DDT and TD conceived the study. All authors were involved in data interpretation. DDT and TD wrote the manuscript with contributions from all other authors.

## Funding

This research was funded by the Deutsche Forschungsgemeinschaft (DFG FOR 1332 to TD and UCM).

## Conflict of Interest Statement

The authors declare that the research was conducted in the absence of any commercial or financial relationships that could be construed as a potential conflict of interest.
